# Longitudinal Assessment of Behaviour and Associated Bio-Markers Following Chronic Consumption of β-Sitosterol β-D-Glucoside in Rats: A Putative Model of Parkinson’s Disease

**DOI:** 10.3389/fnins.2022.810148

**Published:** 2022-02-24

**Authors:** Logan J. Bigelow, Melissa A. Perry, Sarah L. Ogilvie, R. Andrew Tasker

**Affiliations:** ^1^Department of Biomedical Sciences, University of Prince Edward Island, Charlottetown, PE, Canada; ^2^Translational Neuropsychiatry Unit, Department of Clinical Medicine, Aarhus University, Aarhus, Denmark

**Keywords:** BSSG, neurotoxicity, animal model, Parkinson’s disease, rat, neurodegeneration

## Abstract

The consumption of cycad (*Cycas circinalis*) seeds has been linked to the development of Amyotrophic Lateral Sclerosis-Parkinsonism Dementia Complex (ALS-PDC) in humans. ALS-PDC is a clinically variable disease presenting as a combination of symptoms typical of PD and/or ALS. Chronic consumption of β-sitosterol β-D-glucoside (BSSG), a component of the cycad seed, by rats (*Rattus norvegicus*) has been previously reported to initiate a progressive pathology that develops over several months and manifests as behavioural and histopathological changes that resemble characteristic features of Parkinson’s disease. As part of an independent multi-site validation study, we have tried to replicate and further characterize the BSSG model with a focus on motor function, and associated immunohistochemical markers. Beginning at 3 months of age, male CD^®^ (Sprague Dawley) rats (*N* = 80) were dosed orally with either a flour pellet or a flour pellet containing BSSG (3 mg) daily (5×/week) for 16 weeks consistent with previous reports of the model. Following BSSG intoxication, separate cohorts of animals (*n* = 10/treatment) were exposed to a behavioural test battery at 16, 24, 32, or 40 weeks post-initial BSSG feeding. The test battery consisted of the open field test, cylinder test, and ultrasonic vocalization (USV) assessment. No changes in behaviour were observed at any time point. Following behavioural testing, animals were processed for immunohistochemical markers of substantia nigra integrity. Immunohistochemistry of brain tissue revealed no differences in the microglial marker, Iba1, or the dopaminergic integrity marker, tyrosine hydroxylase (TH), in the substantia nigra at any assessment point. The absence of any group differences in behaviour and immunhistochemistry indicates an inability to replicate previous reports. Further investigation into the sources of variability in the model is necessary prior to further utilization of the BSSG model in preclinical studies.

## Introduction

Parkinson’s disease (PD) is a progressive neurodegenerative disorder that affects approximately 100,000 Canadians (Gaskin et al., 2017) and 6 million people worldwide (Dorsey et al., 2007; Vos et al., 2017), making it the second most common neurodegenerative disorder following Alzheimer’s Disease (AD) (de Lau and Breteler, 2006). PD is most well-known for its effects on movement; the early motor symptoms include bradykinesia (a paucity and slowness of movement), muscular rigidity and tremor (Moustafa et al., 2016). In almost all patients, motor symptomology begins on one side of the body, becoming bilateral later in disease (Pagano et al., 2016). In the late stages, motor function is severely impaired, with additional problems related to balance and gait (Boonstra et al., 2008). These disrupted motor functions have additional implications in essential functions such as the production of speech (Tjaden, 2008), among others (Moustafa et al., 2016). The origins of motor dysfunction in PD are largely attributable to the progressive degeneration of dopaminergic neurons in the substantia nigra, a process closely associated with inflammation (H. Wilms et al., 2007). There is currently no cure or means of slowing the progression of PD (Stoker et al., 2018).

In order to improve diagnostics and gain a better understanding of PD, it is necessary to have representative systems that can be observed longitudinally and are amenable to manipulation. This type of study most often involves using animal models. Unfortunately, current models of PD are not progressive and lack several pathological and behavioural hallmarks that can be reliably measured and tracked (Bové et al., 2005; Duty and Jenner, 2011); these features are critical in understanding PD development and how therapies initiated at different time points in disease affect their trajectory. Most importantly, the animal models used need to be reproducible. With excessive variability in outcome, it is impossible to draw conclusion about the effect of interventions (Denayer et al., 2014).

β-sitosterol β-D-glucoside (BSSG) is a component of cycad (*Cycas circinalis*) seeds, consumed by the Chamarro people of Guam (Steele and Guzman, 1987). Consumption of the seeds of the cycad has been linked to the development of Amyotrophic Lateral Sclerosis-Parkinsonism Dementia Complex (ALS-PDC), a disease exhibiting characteristics common to PD and/or ALS (Whiting, 1963; Ince and Codd, 2005). Previous animal studies have indicated that BSSG is a likely culprit in cycad intoxication (Khabazian et al., 2002; Shaw et al., 2002; Tabata et al., 2008). Previous attempts at modelling PD with BSSG describe a next generation animal model, in terms of incorporation of hallmark features (Van Kampen et al., 2014, 2015). Collectively, these studies describe a model that exhibits fundamental behavioural symptoms, both motor and cognitive, as well as associated cellular pathologies inherent to PD. Development of the BSSG model is reportedly progressive and occurs over multiple months in a stereotypical pattern (Braak and Del Tredici, 2017). In association with pathological findings in the brain, characteristic motor and non-motor symptoms develop. Early pathological markers of the disease such as inflammation of the substantia nigra, among others, are unilateral, progressing to become bilateral. Inflammation in the substantia nigra is closely followed by alterations in the expression of typical dopaminergic markers including tyrosine hydroxylase (TH). The onset of motor symptoms coincides with the indicated changes in substantia nigra integrity (Van Kampen et al., 2015).

Considering the promise of the BSSG model, we sought to replicate behavioural and immunohistochemical findings previously observed in the model and to further characterize the model through the addition of the cylinder test and USV assessment, as well as modification of open field parameters and by increasing the number of assessment time points.

## Materials and Methods

### Experimental Animals

All animals used in the experiments were male CD^®^ (Sprague Dawley) rats (*n* = 80) acquired from Charles River Laboratories (Saint-Constant, Quebec). There were 10 control and 10 treatment animals at each of 4 time points (16, 24, 32, 40 weeks), unless otherwise stated. The number of animals required per group was determined by previous power analysis (Van Kampen et al., 2014, 2015). Rats were approximately 3 months of age (200–225 g) at the time of arrival in the animal facility. Upon arrival, cages were randomly assigned to treatment or control. All animals were allowed at least 1 week of acclimation to the facility prior to any manipulations. One week prior to any experimentation, all animals were handled daily for 5 min each by all individuals involved in behavioural assessment. All behavioural testing was conducted between 0800 and 1800 h, with an effort to keep timing as consistent as possible. Behavioural testing was conducted, and outcome assessed, by investigators who were blind to experimental conditions. All procedures were conducted in accordance with the guidelines of the Canadian Council on Animal Care and were approved in advance by the University of Prince Edward Island Animal Care Committee. Animals were maintained under UPEI ACC protocol 16-008. The described procedures were conducted with closest possible adherence to ARRIVE guidelines (Kilkenny et al., 2010).

### Animal Husbandry

Animals were housed in pairs in 50.8 cm × 40.6 cm × 21.6 cm (L × W × H) polycarbonate cages (Ancare Corp., Bellmore, NY, United States). Each cage was equipped with two water bottles, a PVC tube, and a 11.4 cm × 3.8 cm (L × W) nylabone (BioServ, Flemington, NJ, United States) for enrichment. Cages were bedded with Hardwood Beta Chips (North Eastern Products, Warrensburg, NY, United States) and were changed twice a week. The room temperature was maintained between 19 and 22°C, and humidity at 30–52%. The colony room was lit using overhead fluorescent lighting at 300–400 lux, although this was reduced to approximately 150 lux when the animals were in the housing rack. No animals were placed on the top shelf of the rack system and so were constantly protected from direct light. The housing room was on 12-h light-dark cycle with the lights coming on at 0600 and going off at 1800. When it was necessary to enter the room during the dark cycle, a red lamp was used to provide the necessary light. The ambient room sound was 70–80 Db, with most of the sound in the room emanating from the animal transfer station which operated 24 h. All animals were maintained on an *ad libitum* diet of Laboratory Rodent Diet 5001 (LabDiet, Saint Louis, MO, United States) and tap water, unless otherwise stated. During the intoxication period, the animals’ location in the rack was randomized 5×/week. Following the intoxication period, the animals’ location was randomized once a week following weekly weighing. The primary experimenter was male.

### Pellets

Flour pellets and BSSG-laced flour pellets were obtained from Neurodyn Life Sciences Inc. (Charlottetown, PE, Canada). Pellets, once received, were kept at −20°C until 2 h prior to use, when they were removed from the freezer and left at room temperature. Any extra pellets remaining from any one day would be stored at room temperature and used the following day.

Pellets contained distilled/deionised water, flour, red dye (BSSG), green dye (flour), banana flavouring, and in the BSSG pellets, 3 mg of BSSG. In preparation, the ingredients were combined and the resultant dough was homogenized, pressed, cut and weighed. The pellets were then dried in an oven at 65°C overnight. After cooling to room temperature, the pellets were weighed again, vacuum sealed and stored at −20°C until shipping. Dried pellets measured 1–2 cm in diameter and weighed approximately 1 g. BSSG was synthesized from stigmasterol and has previously been determined to be 95–97% pure (Van Kampen and Robertson, 2017).

### β-Sitosterol β-D-Glucoside Feeding

**FIGURE 1:**
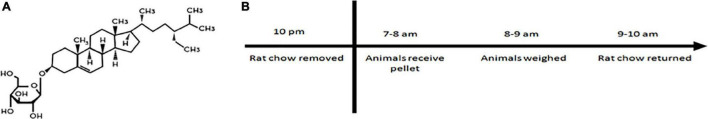
**(A)** Structure of β-sitosterol β-D-glucoside (BSSG). **(B)** Daily feeding schedule for BSSG administration.

Beginning at ∼3 months of age, rats were initiated on a 16 week intoxication where they received either a flour pellet or flour pellet laced with BSSG (3 mg) (Figure 1A). The pellet was administered once a day, 5×/week (Monday–Friday). In order to ensure that the animal would consume the pellet and for maximal absorption with minimal gut competition (Van Kampen et al., 2015), access to food was revoked 12 h prior to pellet administration and 2 h afterward (Figure 1B). During the weekend, animals were given *ad libitum* access to food from 2 h post-feeding on Friday to 12 h before pellet administration on Monday morning. To prevent rats from consuming their cage mate’s pellet, a Plexiglas divider was inserted into the center of the cage separating the animals until they had fully consumed their respective pellets. Animals had approximately 30 min to consume the pellet. Prior to removing the divider, the bedding of the cage was searched in case the animal had buried the pellet. If the animals did not consume the pellet, the pellet was brought to their attention by moving it toward their face; the animal was then allowed an additional 10 min to consume the pellet. It was rare that an animal did not consume the pellet during the first 30 min, and all animals consumed the pellet when given a second opportunity. After confirming the pellet was consumed, the animals were weighed and returned to their home cage. Animals were placed back into the rack in a random location in order to control for any effects related to position.

### Behavioural Testing

Following intoxication, cohorts of animals were tested in the open field arena, cylinder test and assessed for USV content at 16, 24, 32, and 40 weeks post-initiation of BSSG feeding. Animals were euthanized following the completion of the behavioural testing series at each assessment point.

#### Open Field

The open field arena measured 90.2 cm × 90.2 cm × 61 cm (L × W × H) and was constructed with black corrugated plastic. The floor of the arena was also constructed of black corrugated plastic (KwikKopy, Charlottetown, PE, Canada) which was situated on top of a plastic pallet which kept the area level. In opposite corners of the room, two lamps with 40-watt bulbs were positioned toward the wall to avoid shadows within the arena. The animal was moved from the colony room to the behavioural testing suite in a holding cage. The holding cage was a small rat cage with the bedding removed and a strip of brown paper towel placed in the bottom. In the testing suite, the animal was removed from the holding cage and placed in the maze. All animals were placed in the maze from the same location, facing the same direction (center of the arena). The animal was left to freely explore the maze for one hour in the absence of the experimenter while behaviour was video recorded. Following the hour, the animal was removed from the maze, a fecal count was taken, the maze was cleaned with 70% alcohol, and the animal was returned to the colony room. To ensure the alcohol was completely evaporated from the maze prior to the next testing session, a 10 min interval was used between animals. Videos were later assessed for measures of distance travelled.

#### Cylinder Test

A clear polycarbonate cylinder measuring 26.7 cm in diameter and 46.4 cm high was used. The cylinder was placed on top of a sheet of black corrugated plastic to provide contrast, and a camera was mounted on a tripod above the cylinder. The animal was taken from the colony room to the testing suite in a holding cage. In the testing suite, the animal was removed from the cage, placed into the cylinder and behaviour was video-recorded for 10 min in the absence of the investigator. Following the 10 min recording period, the animal was removed from the cylinder, a count of the fecal matter within the cylinder was made and the cylinder was cleaned with Accel TB (0.5 % hydrogen peroxide) (Virox Technologies Inc., Oakville, ON, Canada). The animal was then returned to the colony room. The primary measures of interest in the cylinder test were laterality of limb use, defined as the absolute value of % left paw placements–% right paw placements, number of rears, defined as the number of times the animal’s forelimbs break contact with the bottom of the cylinder, and total number of paw placements on the cylinder wall.

#### Ultrasonic Vocalization Recordings

Cage mates were placed in a 50.8 cm × 40.6 cm × 21.6 cm (L × W × H) polycarbonate arena (Ancare Corp., Bellmore, NY, United States). The arena was identical to the housing cages with the exception of the bedding, tunnel, nylabone and water bottles removed. The animals were brought to the behavioural testing suite in individual holding containers lined with a strip of brown paper towel. The animals, who were cage mates, were then placed in the testing arena together, and the lid was secured. The USV microphone (Avisoft Bioacoustics, Glienicke/Nordbahn, Germany) was placed over the center of the cage, approximately 30.5 cm from the lid. The animals were recorded for 10 min. Following the recording, the animals were returned to their holding cages and returned to the housing unit. Due to the negligible number of 22 kHz calls, the primary measure of interest was number and duration of 50 kHz calls.

#### Behavioural Analysis

Open field data was automatically scored *via* ANY-maze^®^ behavioural tracking software (Stoelting Company, Wood Dale, IL, United States). The cylinder test was scored manually using ANY-maze. In both cases, the experimenters were blind to experimental group. All behavioural testing procedures, with the exception of cylinder testing, were video recorded using an overhead Logitech C310 webcam (Logitech, Newark, CA, United States) at 720 p resolution. For the cylinder test, either a Canon HD CMOS 32× (Cannon Canada, Brampton, ON, Canada), or a JVC Everio (JVC Canada, Toronto, ON, Canada) mounted to a tripod was used (recording was at 480 p resolution). All behavioural testing was video recorded and stored regardless of whether it was scored in real time. USV analysis was performed using DeepSqueak 2.6.2 (Coffey et al., 2019). Analysis of 50 kHz calls was scored using the short rat call neural network.

### Immunohistochemistry

#### Tissue Processing

Euthanasia occurred approximately 1 week after the completion of behavioural testing. Animals were anesthetized with isoflurane prior to transcardial perfusion, initially with saline, followed by 4% paraformaldehyde. The brains were post-fixed in 4% paraformaldehyde for 24 h and then transferred to 30% sucrose. A needle was pierced through the right side of the brain to identify hemispheres. Whole brains were sectioned on a vibratome (Vibratome 1000 Plus, Leica Biosystems, Concord, ON, Canada) at 40 μm and stored in Millonig’s phosphate-buffered saline at 4°C until later use.

At the time of use, tissue was washed 3× for 10 min each in Tris-Buffered Saline (TBS) (VWR, Edmonton, AB, Canada) and then incubated in 2× Saline Sodium Citrate at 65 °C for 20 min. Following incubation, the sections were washed 2× in TBS for 10 min each and then blocked for 1 h in 2% donkey serum (Sigma-Aldrich, Oakville, ON, Canada) dissolved in TBS. Post-blocking, primary antibodies, goat anti-Iba1 (NOVUS, Toronto, ON, Canada, 1:2000) and mouse anti-TH (EMD Millipore, Oakville, ON, Canada, 1:10000) in 5% donkey serum and 0.3% TBS triton (VWR, Edmonton, AB, Canada) were applied to the sections and left overnight. The following day, the sections were washed 3× in TBS for 10 min each. Fluorescent secondary antibodies, donkey anti-mouse (488 nm) (Life Technologies, Thermo Fisher Scientific, Ottawa, ON, Canada) and donkey anti-goat (594 nm) (Life Technologies, Thermo Fisher Scientific, Ottawa, ON, Canada) were applied at a concentration of 1:600 in phosphate-buffered saline (PBS). The plates were then wrapped in aluminium foil and left for 4 h. The tissue was then washed 3× in TBS at 5 min per wash and transferred to Millonig’s phosphate-buffered saline (in-house) until mounting. The sections were then mounted using Fluoromount-G (Thermo Fisher Scientific, Ottawa, ON, Canada). Relevant sections with no primary antibody applied were used as controls. The experimenters were blind to treatment conditions.

#### Tissue Imaging

All mounted tissue was cured for at least one week prior to being imaged. Images were taken on a Zeiss Observer Z1 microscope (Zeiss Canada, Toronto, ON, Canada) at 200× magnification. Alexa Fluor 594 and Alexa Fluor 488 wavelength filters were used to capture Iba1 and TH, respectively. Exposure for each channel was regularly optimized to provide the best quality images for cell counts.

**FIGURE 2:**
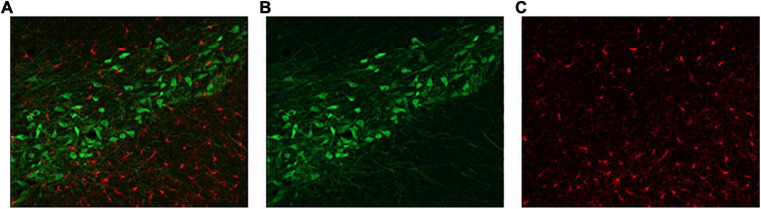
Representative substantia nigra sections. **(A)** Double-labelled sections of interest were assessed for **(B)** TH-(green) and **(C)** Iba1-positive cells (red).

Three sections of the substantia nigra, taken from each hemisphere of the brain (*n* = 6), were selected to represent rostral, middle, and caudal sections (Figure 2). Determining the location of the sections of interest was done by preselection of a section based on comparison of gross appearance and rostro-caudal gradient (Paxinos and Watson, 2007). The distinct pattern of the substantia nigra under the microscope was also used to confirm section suitability. One image was taken per side of the brain. The precise location of the image was one field of view from the first indications of the beginning of the substantia nigra. Coronal images were taken at 0.54 μm through the tissue, stacked, and maximally exposed using Zen Pro (Zeiss Canada, Toronto, ON, Canada). Images for Iba1 and TH were taken simultaneously. The experimenters were blind to conditions.

#### Counting Methods

Using Zen 2011, Blue Edition (Carl Zeiss Canada Ltd., Toronto, ON, Canada), the substantia nigra was outlined using TH cells as a guide. Iba1 cells were counted within that area. To account for the area size, cell counts were determined as a proportion of the area. For TH, the substantia nigra was outlined. An intensity measurement was determined for each section. Due to a lack of difference in intensity measurements across hemispheres, the intensities for the six sections were averaged for each animal. Three background intensity measurements were taken using standardized coordinates and averaged. The final intensity measurement was corrected for both background intensity and area size. For each rat there were a total of 6 sections (3 per hemisphere). Due to a lack of differences between hemispheres the counts or intensity for the six sections was averaged for each rat.

### Statistical Analysis

All statistical analysis was performed using IBM SPSS Statistics 22 (IBM Canada, Markham, ON, Canada). All graphs were constructed using Prism 5 (GraphPad, La Jolla, CA, United States). Results are presented as mean ± SEM unless otherwise stated. When comparing two or more sections in cell counting, a one-way analysis of variance (ANOVA) was used. For all other outcome assessments, a two-tailed unpaired *t*-test was performed. Significance was indicated by a *p* < 0.05.

## Results

### Open Field

**FIGURE 3:**
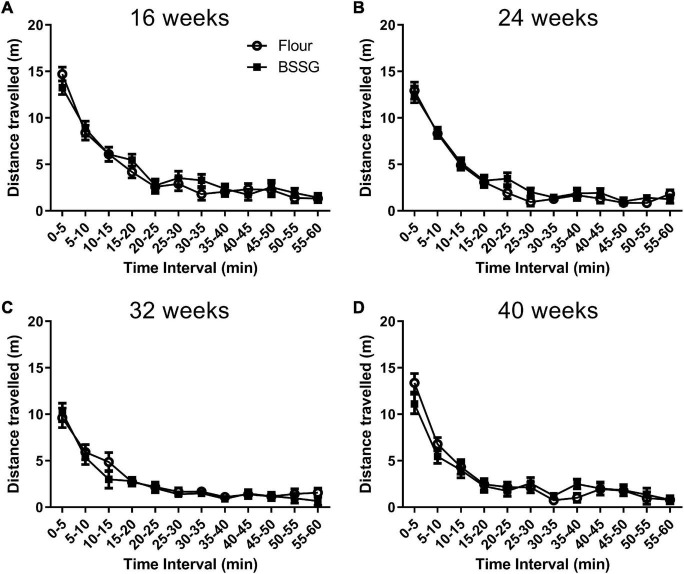
Distance travelled in the open field in 5 min intervals. **(A)** 16 weeks; **(B)** 24 weeks; **(C)** 32 weeks; **(D)** 40 weeks. Data are shown as means ± SEM, *n* = 8–10.

The primary measure of interest in the open field was distance travelled. When observing the distance travelled in the open field arena in 5 min intervals, it was found that while animals in the 16 week, [*F*(11,216) = 62.32, *p* < 0.0001], 24 week, [*F*(11,216) = 88.64, *p* < 0.0001], 32 week, [*F*(11,204) = 37.49, *p* < 0.0001], and 40 week, [*F*(11,168) = 47.69, *p* < 0.0001], groups travelled less as the trial proceeded, there were no treatment × time interactions at the 16 week, [*F*(11,216) = 0.68, *p* = 0.758], 24 week, [*F*(11,216) = 0.55, *p* = 0.871], 32 week, [*F*(11,204) = 0.51, *p* = 0.895], or 40 week, [*F*(11,168) = 1.0, *p* = 0.439], observation points (Figure 3).

**FIGURE 4:**
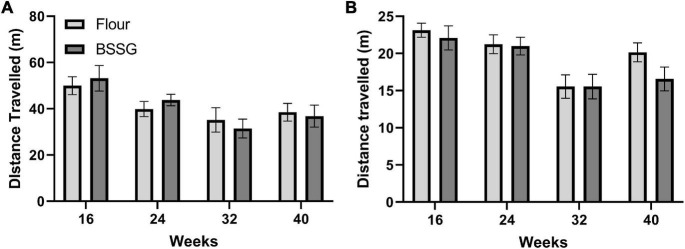
Distance travelled in the open field. **(A)** 60 min duration and **(B)** first 10 min within the open field arena. There were no group differences in distance travelled at any of the assessment points. Data are shown as means ± SEM, *n* = 8–10.

No treatment group differences were observed in distance travelled over one hour in the open field for the 16 week, [*t*(18) = 0.480, *p* = 0.637], 24 week, [*t*(18) = 0.959, *p* = 0.353], 32 week, [*t*(17) = -0.568, *p* = 0.577], or 40 week [*t*(15) = -0.278, *p* = 0.785], assessment points (Figure 4A). Considering most of the movements occurred when the animal was first placed into the open field arena (see Figure 3), the first 10 min of the open field test was examined separately. Observation of the first 10 min of distance travelled in the open field test revealed no differences between groups at 16 weeks, [*t*(18) = 0.544, *p* = 0.593], 24 weeks, [*t*(18) = 0.138, *p* = 0.892], 32 weeks, [*t*(17) = 0.0100, *p* = 0.992], or 40 weeks, [*t*(15) = -1.77, *p* = 0.0960] (Figure 4B).

### Cylinder Test

**FIGURE 5:**
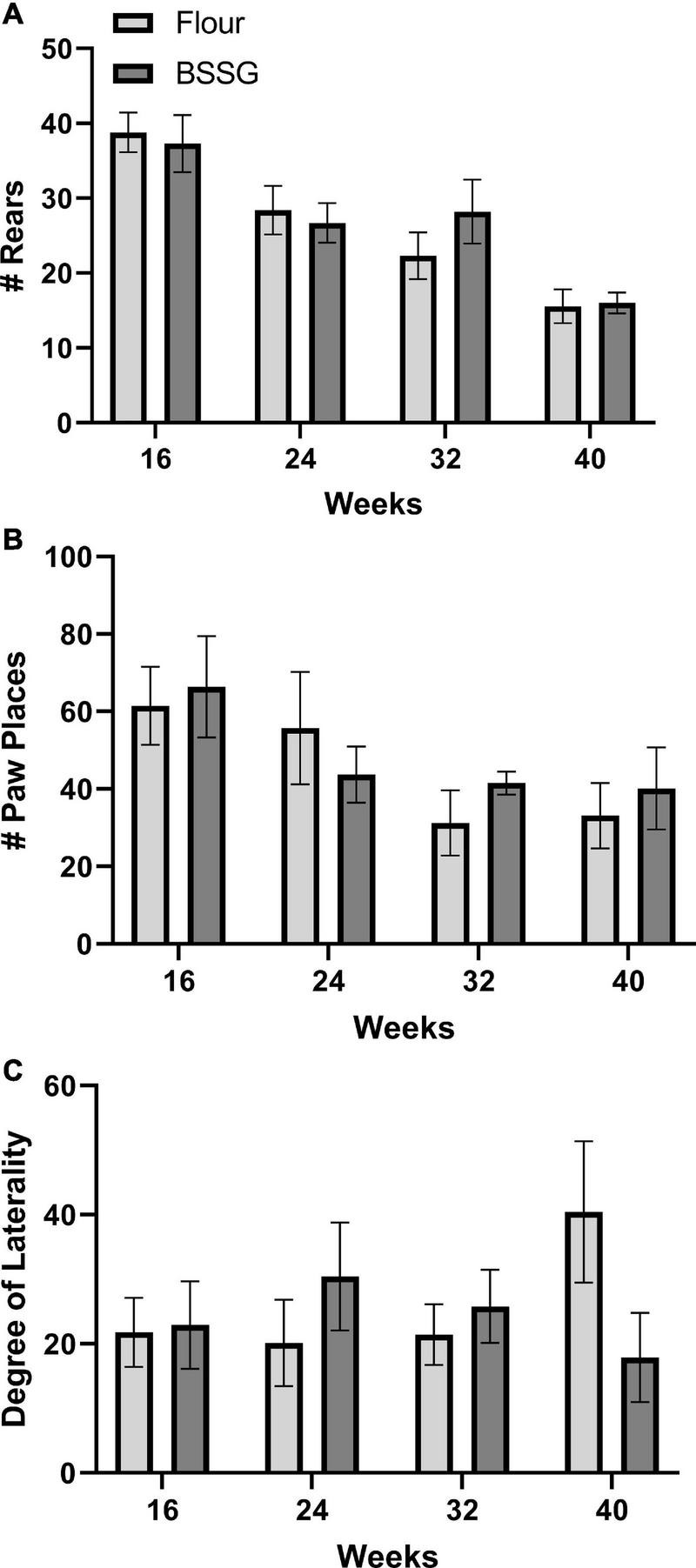
Cylinder test outcomes. **(A)** Total number of rears. **(B)** Total number of paw placements. **(C)** Degree of laterality of paw placements. Data are shown as means ± SEM, *n* = 8–10.

There were no group differences in the number of rears in the cylinder at the 16 week, [*t*(17) = 0.312, *p* = 0.759], 24 week, [*t*(18) = 0.406, *p* = 0.689], 32 week, [*t*(18) = -1.12, *p* = 0.279], or 40 week, [*t*(15) = 0.163, *p* = 0.873], assessment points (Figure 5A). Similarly, there were no group differences in number of paw placements on the wall for the 16 week, [*t*(17) = 0.295, *p* = 0.771], 24 week, [*t*(18) = 1.15, *p* = 0.265], 32 week, [*t*(18) = 0.741, *p* = 0.468], or 40 week, [*t*(15) = 0.524, *p* = 0.608], groups (Figure 5B). There were also no group differences in laterality of limb placement in the cylinder at 16 weeks, [*t*(17) = 0.132, *p* = 0.897], 24 weeks, [*t*(18) = -0.960, *p* = 0.350], 32 weeks, [*t*(18) = 0.591, *p* = 0.562], or 40 weeks, [*t*(15) = -1.69, *p* = 0.111] (Figure 5C).

### Ultrasonic Vocalizations

**FIGURE 6:**
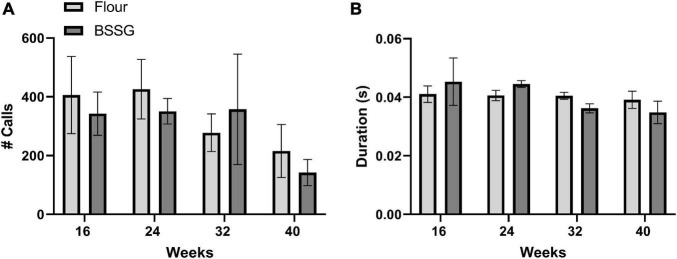
Assessment of USVs. **(A)** Total number of USVs and **(B)** Duration of USVs. Data are shown as means ± SEM, *n* = 4–5.

Measurement of both the number and duration of USVs also revealed no differences between BSSG and control treated rats. There were no significant group differences for the number of calls at 16 weeks, [*t*(8) = 0.421, *p* = 0.685], 24 weeks, [*t*(8) = 0.683, *p* = 0.683], 32 weeks, [*t*(6) = 0.402, *p* = 0.702], or 40 weeks, [*t*(6) = 0.732, *p* = 0.492] (Figure 6A). There were also no significant group differences for the duration of calls at 16 weeks, [*t*(8) = 0.495, *p* = 0.634], 24 weeks, [*t*(8) = 1.85, *p* = 0.101], 32 weeks, [*t*(6) = 2.18, *p* = 0.0724], or 40 weeks, [*t*(6) = 0.888, *p* = 0.409] (Figure 6B).

### Immunohistochemistry

**FIGURE 7:**
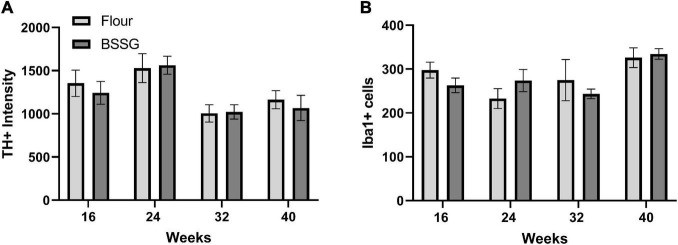
Immunohistochemistry results. **(A)** Tyrosine hydroxylase (TH) immunofluorescence intensity and **(B)** Iba1 cell counts. Data are shown as means ± SEM *n* = 7–10.

To determine if BSSG intoxication resulted in any changes in PD-relevant markers of dopaminergic function and inflammation we measured the expression of TH and Iba1. There were no group differences for total TH counts in the substantia nigra at 16 weeks, [*t*(18) = 0.541, *p* = 0.595], 24 weeks, [*t*(18) = 0.178, *p* = 0.861], 32 weeks, [*t*(17) = 0.131, *p* = 0.897], or 40 weeks, [*t*(14) = 0.552, *p* = 0.589] (Figure 7A). Further, there were no group differences for Iba1 intensity in the substantia nigra at 16 weeks, [*t*(18) = 1.41, *p* = 0.174], 24 weeks, [*t*(18) = 1.20, *p* = 0.244], 32 weeks, [*t*(17) = 0.682, *p* = 0.504], or 40 weeks, [*t*(14) = 0.305, *p* = 0.765] (Figure 7B).

## Discussion

There were no group differences in behaviour or immunohistochemistry at any of the four assessment points. These findings stand in stark contrast to prior reports of the BSSG model in rats, which revealed progressive onset of motor and cognitive deficits accompanied by significant changes in associated immunohistochemical markers (Van Kampen et al., 2014, 2015). The reason(s) for these discrepancies are unclear with the most likely candidates being some aspect of the methodology, the genetics/husbandry of the rats or the integrity/bioavailability of BSSG.

Previously, the open field was used successfully to detect progressive motor deficits that were responsive to levodopa following BSSG intoxication (Van Kampen et al., 2015). The open field is relatively easy to perform and considering there was a pre-established reference, interpretation of findings would be more straightforward and so the paradigm was considered a non-labour-intensive behavioural feature to replicate as part of model validation. The cylinder test had not been previously used in the BSSG model, but based on prior observations and published reports of laterality in substantia nigra markers in the model (Van Kampen et al., 2014), we hypothesized that there may be differences in limb use laterality in the earlier stages of disease development. This laterality effect could possibly be replaced by a bilateral akinetic effect in later stages as laterality in nigrostriatal markers subside. The akinetic effect could be observed through number of rears and total number of paw placements. Like the open field, the convenience of the cylinder test resides in its ease of use and low equipment demands, and we and others have used this test previously in studies of unilateral ischemic stroke (Schallert et al., 2000; Adkins et al., 2004; Hume and Tasker, 2020) as well as parkinsonism (Schallert et al., 2000). This study is the first time USV content was assessed in the BSSG model. Given that PD often affects the ability to produce speech, initially as a result of nigrostriatal deterioration (Pinto et al., 2004), we hypothesized to observe a decrease in both the duration and number of calls.

The associated immunohistochemical markers, Iba1 and TH, were chosen because both have been previously reported to show abnormalities in the substantia nigra early in disease development. Previously, differences in Iba1 in the substantia nigra were present at the 16 week assessment point and differences in TH at the 24 week assessment point (Tabata et al., 2008; Van Kampen et al., 2014). In the current study, there were no significant group differences at any of the observed time points for either marker indicating a lack of between-study congruence.

The BSSG model has been utilized on two separate occasions without issue (Tabata et al., 2008; Van Kampen et al., 2014). The methods employed in all of the previous studies were similar (or identical) to those in the current study. The strain and sex of animals varied between those two previous studies of the BSSG model; however, the current study used male CD rats which are the same as those used by Van Kampen et al. (2015). The closely related SD strain had also been previously used with positive results, although this was in female animals (Tabata et al., 2008). Considering previous success with the strain used in the current study (CD), it is unlikely that strain is responsible for the lack of findings in our study. Animal housing, husbandry and treatment protocols in the current study were also similar but not identical to those previously described (Tabata et al., 2008; Van Kampen et al., 2014). In the current study, animals were housed together since the time of their arrival; however, animals were singly housed in Van Kampen et al. (2015). It is known that animals housed individually exhibit altered behavioural and biochemical markers compared to group-housed animals (Pérez et al., 1997; Pinelli et al., 2017). The intoxication regiment was essentially identical in the current study to that described in previous publications of the BSSG model. The only difference between the intoxication procedure was a longer food deprivation prior to pellet administration in the previous study (Van Kampen et al., 2014) (∼16 h) compared to the current study where a shorter period of food restriction (12 h) was implemented as directed by our institutional animal care and use committee. The BSSG content/pellet, length of the intoxication regiment, intoxication days per week, and time between receiving pellet and return of food were the same. Although the described differences between studies may certainly introduce some variability, it is not convincing that any of these factors alone, or in conjunction, could be responsible for such a dramatic disruption at both the behavioural and immunohistochemical level. These conclusion reinforce the notion that there was an issue with the BSSG pellets.

As stated previously, the BSSG pellets were obtained from an external source that was consistent with the previous reports (Tabata et al., 2008; Van Kampen et al., 2014), although the sources of raw materials may have differed. The BSSG pellets were made by combining deionized/distilled water, flour, red or green dye, banana flavour, and newly synthesized BSSG. The ingredients were homogenized, cut into pellets, dried at 65°C overnight, vacuum sealed and stored at −20°C until use. This is the same process that was used previously without any issue. To determine if the BSSG pellets were the source of the problem, confirmation of the purity of the BSSG and conduction of a bioavailability study comparing our pellets with those of Van Kampen et al. (2014, 2015) is warranted. These studies are currently in progress.

Our current understanding of PD as a disease has gone through dramatic changes and restructuring over the past couple of decades. It is an exciting time to be studying the disease, with new avenues of investigation apparent. While these landmark discoveries bring researchers closer to understanding, and hopefully curing PD, they also reinforce the complexity of Parkinson’s disease and Parkinsonism that is not fully reflected in currently established animal models. There is an urgent need for new and improved animal models in PD research. Prior BSSG modelling of PD in rats has provided evidence that the model encompasses many of the hallmark features of PD but could not be replicated in the current study. Consequently, further investigation of the BSSG model is required because the previously described power and potential benefits of the model merits further inspection.

## Data Availability Statement

The raw data supporting the conclusions of this article will be made available by the authors, without undue reservation.

## Ethics Statement

The animal study was reviewed and approved by University of Prince Edward Island Animal Care Committee.

## Author Contributions

RT: funding acquisition, methodology, project administration, resources, supervision, and reviewing and editing of manuscript. MP and SO: data acquisition, data analysis, and reviewing and editing of manuscript. LB: data acquisition, data analysis, and writing original draft. All authors contributed to the article and approved the submitted version.

## Conflict of Interest

The authors declare that the research was conducted in the absence of any commercial or financial relationships that could be construed as a potential conflict of interest.

## Publisher’s Note

All claims expressed in this article are solely those of the authors and do not necessarily represent those of their affiliated organizations, or those of the publisher, the editors and the reviewers. Any product that may be evaluated in this article, or claim that may be made by its manufacturer, is not guaranteed or endorsed by the publisher.
